# Developments toward the Implementation of ^44^Sc Production at a Medical Cyclotron

**DOI:** 10.3390/molecules25204706

**Published:** 2020-10-14

**Authors:** Nicholas P. van der Meulen, Roger Hasler, Zeynep Talip, Pascal V. Grundler, Chiara Favaretto, Christoph A. Umbricht, Cristina Müller, Gaia Dellepiane, Tommaso S. Carzaniga, Saverio Braccini

**Affiliations:** 1 Laboratory of Radiochemistry, Paul Scherrer Institute, 5232 Villigen-PSI, Switzerland; 2 Center of Radiopharmaceutical Sciences ETH-PSI-USZ, Paul Scherrer Institute, 5232 Villigen-PSI, Switzerland; rogerhasler26@gmail.com (R.H.); zeynep.talip@psi.ch (Z.T.); pascal.grundler@psi.ch (P.V.G.); chiara.favaretto@psi.ch (C.F.); christoph.umbricht@gmail.com (C.A.U.); Cristina.mueller@psi.ch (C.M.); 3 Albert Einstein Center for Fundamental Physics, Laboratory of High Energy Physics, University of Bern, 3012 Bern, Switzerland; gaia.dellepiane@lhep.unibe.ch (G.D.); tommaso.carzaniga@lhep.unibe.ch (T.S.C.); saverio.braccini@lhep.unibe.ch (S.B.)

**Keywords:** scandium-44, ion exchange chromatography, medical cyclotron

## Abstract

^44^Sc has favorable properties for cancer diagnosis using Positron Emission Tomography (PET) making it a promising candidate for application in nuclear medicine. The implementation of its production with existing compact medical cyclotrons would mean the next essential milestone in the development of this radionuclide. While the production and application of ^44^Sc has been comprehensively investigated, the development of specific targetry and irradiation methods is of paramount importance. As a result, the target was optimized for the ^44^Ca(p,n)^44^Sc nuclear reaction using CaO instead of CaCO_3_, ensuring decrease in target radioactive degassing during irradiation and increased radionuclidic yield. Irradiations were performed at the research cyclotron at the Paul Scherrer Institute (~11 MeV, 50 µA, 90 min) and the medical cyclotron at the University of Bern (~13 MeV, 10 µA, 240 min), with yields varying from 200 MBq to 16 GBq. The development of targetry, chemical separation as well as the practical issues and implications of irradiations, are analyzed and discussed. As a proof-of-concept study, the ^44^Sc produced at the medical cyclotron was used for a preclinical study using a previously developed albumin-binding prostate-specific membrane antigen (PSMA) ligand. This work demonstrates the feasibility to produce ^44^Sc with high yields and radionuclidic purity using a medical cyclotron, equipped with a commercial solid target station.

## 1. Introduction

The concept of theragnostics [1] in nuclear medicine is based on the use of radionuclides of preferably the same element (radioisotopes) to enable the application of chemically identical radiopharmaceuticals for both diagnosis and therapy [2]. The combination of suitable radionuclides is known as “matched pairs” and this can be performed with different radioisotopes of scandium, namely ^44^Sc for diagnosis and ^47^Sc for therapy [3].

The favorable decay properties of ^44^Sc (E_β+av_ = 632 keV, T_1/2_ = 4.04 h, E_γ_= 1157 keV, I = 100%) [4,5] for cancer diagnosis using positron emission tomography (PET) makes it a promising candidate for imaging applications in nuclear medicine [6]. Scandium-44 could be used as an alternative to the currently used ^68^Ga (T_1/2_ = 68.3 min), with a longer half-life which would improve image quality [7,8], when images are recorded at later time points (when tumor-to-background ratios are enhanced) [9]. This is desirable for imaging with radiopharmaceuticals with a longer biological half-life [6]. With chemistry similar to the therapeutic ^90^Y and ^177^Lu, ^44^Sc can be used for diagnosis as well as for the planning, staging, and monitoring of radionuclide therapy. The physical half-life of ^44^Sc also makes it attractive to be produced from a commercial perspective, as it is easier to distribute than the likes of ^68^Ga radiopharmaceuticals [8].

^44^Sc was used extensively in preclinical studies at Paul Scherrer Institute (PSI), Switzerland, using various targeting agents, namely folates [7,8], somatostatin analogs [2] and PSMA-targeted ligands [10].

Cyclotron-produced ^44^Sc was also delivered to the Zentralklinik Bad Berka, Germany, where it was applied to two patients [9,11]. Whole-body PET/CT scans were performed after injection of ^44^Sc-DOTATOC. Patient scans showed excellent uptake in liver metastases of a neuroendocrine tumor, high resolution and excellent contrast, especially at later time points.

While the production and application of ^44^Sc has been comprehensively investigated [7,8,12,13], targetry optimization for this nuclear reaction has been scarcely explored. This novel radionuclide will only have a clinical impact if it can be made available in appropriate quantity and quality. For this reason, the implementation of its production with existing compact medical cyclotrons [14] would mean the next essential milestone in the development of this radionuclide. Along this line, the development of specific targetry and irradiation methods is of paramount importance [15].

The production of ^44^Sc was achieved with a Ca target via the Ca(p,n)^44^Sc nuclear reaction. Several sites used natural Ca [12,16], while the use of enriched ^44^Ca has also been extensively used [8,13,17] and studied [18]. From the published data [19,20,21], it is seen that the production cross section has a broad maximum of ~650 mb in the proton energy range 8–13 MeV. It is important to mention that this corresponds to the characteristic beam energies of compact medical cyclotrons (15–25 MeV). Calcium carbonate targets have predominantly been used for target irradiation. Up to 50 µA beam intensity on target has been reported and high yields (approximately 2 GBq) obtained on ~10 mg enriched target material when irradiating for up to 90 min [8,13]. The use of CaCO_3_ has had its challenges, therefore, a more robust target material, CaO, was envisaged [22].

^44^Sc production methods were first developed at the IP2 irradiation station at PSI [23] using specific research targets. At this facility, the 72 MeV proton beam obtained from the Injector 2 separated sector cyclotron was degraded down to the energy range of interest (~10–13 MeV). The implementation of ^44^Sc production at a compact medical cyclotron using a commercial solid target station represents a challenging, but necessary, step in view of ^44^Sc clinical application. In this article, the methodologies developed are described, as well as the results obtained using the IBA Cyclone 18 MeV proton cyclotron in operation at the Bern University Hospital (Inselspital—hereafter referred to as UniBe), Switzerland. A proof-of-concept preclinical study, using the ^44^Sc produced at the UniBe medical cyclotron, was performed using a previously developed albumin-binding PSMA ligand.

## 2. Results and Discussion

### 2.1. Target Preparation

**Figure 1:**
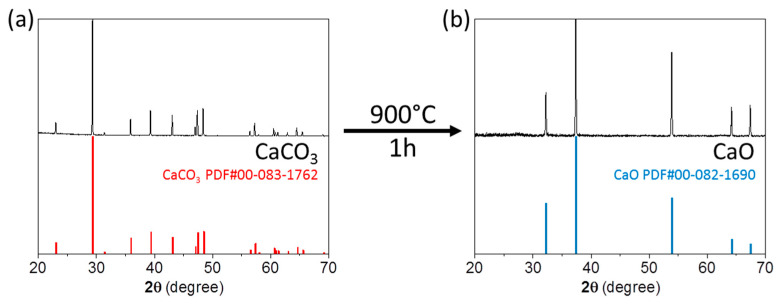
X-ray diffraction (intensity) patterns obtained from commercially available CaCO_3_ (**a**) and the resultant CaO, obtained after thermal decomposition (**b**).

**Figure 2:**
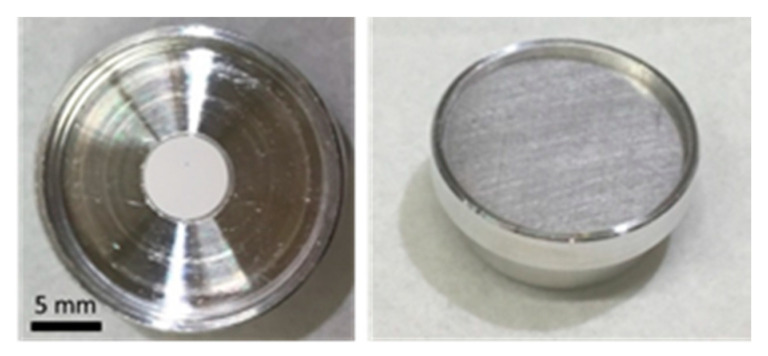
Pressed CaO target (6 mm × 0.5 mm) nestled in the indentation of an Al (99.5% pure) capsule. The capsule was sealed and prepared for irradiation at PSI’s IP2 irradiation station.

CaO was obtained through thermal decomposition of CaCO_3_ at 900 °C. X-ray diffraction (XRD) analysis of the powder obtained showed complete conversion of CaCO_3_ (Calcite, rhombohedral crystal structure, S.G.: R-3c) to CaO (cubic crystal structure, S.G.: Fm3m) (Figure 1). CaO targets were prepared by pressing the CaO powder into disk-shaped pellets, 6 mm in diameter and 0.5 mm thick (Figure 2). Densities of the pressed targets were estimated by the mass to volume ratio. They presented a relative density of ca. 65% of their theoretical value (ρ_CaO_ = 3.34 g/cm^3^ [1]).

The new target design was a necessary step to improve the yield and consistency of the production, as the previous target was rudimentary and had poor homogeneity. As a result, there was no consistency as to where the proton beam hit the target material, or whether some of the target material was not irradiated at all. Taking practical issues into consideration, including the cost of target material, a target of 6 mm in diameter and 0.5 mm thickness was considered to be the best option. This would produce a target about 30 mg in mass which along with the increase in Ca^2+^ ions with conversion to oxide, would increase the yield of product of a factor ~1.7 for the same irradiated mass or ~2.2 for the same irradiated volume. These improvements can be considered a fundamental step toward the implementation of a standard production procedure. The use of carbonate as a pellet presented clear disadvantages, in that the encapsulated PSI target would swell on occasion, while at UniBe these targets resulted in degassing upon irradiation (Supplementary Materials). To cope with the severe radiation protection rules in a hospital-based environment, the beam intensity had to be decreased to such a low value that it made the production practically unfeasible. Conversion to oxide targets not only improved the yield, but also solved these issues, leading to a robust and stable target for a reliable and safe production from a radiation protection perspective (Figure 2).

To avoid the formation of calcium hydroxide (Ca(OH)_2_) or CaCO_3_, the prepared CaO powder and the pressed targets were stored under Ar atmosphere (or in a desiccator under vacuum) until use. The XRD study (Supplementary Materials, Figure S1) indicated that the pressing of targets under non-inert conditions was possible, on the proviso that exposure to the elements was kept to a minimum.

While the oxide targets were found to be an improvement over its carbonate predecessor, they still had to be handled carefully to ensure optimum results. The targets remain hygroscopic over time and it is recommended that the target be prepared as soon as possible before encapsulation and irradiation. At PSI, the encapsulated targets could hold for a maximum period of one week without further treatment; however, the magnetic coin system at UniBe was challenging and special precautions were needed to be taken in this regard. Once the target was prepared, it was necessary to vacuum pack each one individually and store in a desiccator containing potassium hydroxide or under inert gas. Disastrous results were observed when such measures were not taken in the preparation of targets, or when the prepared gas-filled envelope was damaged (Supplementary Materials, Figure S4). It is also important to note that the insertion of the target coin in the target station required care, to minimize the exposure of the target to traces of moisture. The moisture in the air may react with the CaO target to form CaCO_3_, making the pellet increase in volume and the target stick in the coin. The envelope containing the target coin had to be opened just before insertion into the target station and the piston to be activated immediately to ensure pressing the coin with the O-ring. If CaCO_3_ would form in the target inserted in the station, the increase of volume could possibly cause the coin to get stuck inside the station, with severe radiation protection consequences. In particular, a manual intervention would be required, with possible surface contamination due to the opening of the target coin. The installation of the coin occurred the evening before the irradiation (as the cyclotron was utilized in the early morning), such that the dose received by the operators was minimized.

### 2.2. Irradiation Conditions

The 6 mm ^nat^CaO targets were irradiated at PSI’s IP2 irradiation station, using the 72 MeV proton beam from Injector 2, for periods from 0.5 h to 1.5 h to test the robustness of the material under stringent conditions. A 3.4 mm Nb disc was used as degrader to decrease the proton energy to the desired energy window [23], to ensure maximum ^44^Sc production via the ^44^Ca(p,n)^44^Sc nuclear reaction. The optimal beam current, with regard to dissolution properties of the irradiated target in 1.0 M HNO_3_, was found to be at 45–50 µA. After optimizing the irradiation conditions for the proton beam at PSI, enriched ^44^CaO targets were irradiated.

**Figure 3:**
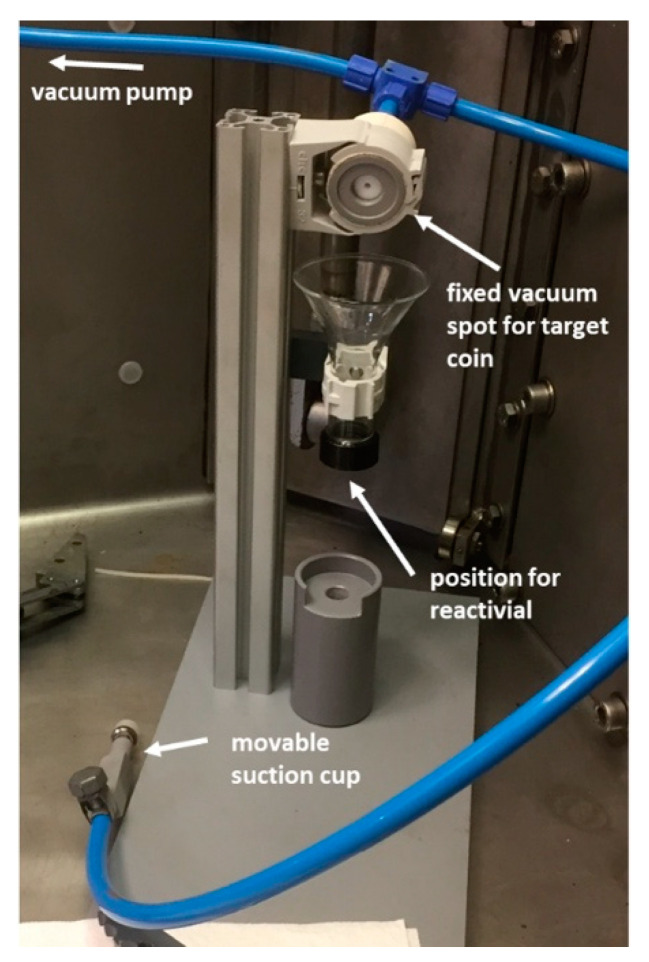
Representative picture of the setup used in the hot cell to open the irradiated targets received from the Bern cyclotron laboratory.

The Al encapsulated target capsules used for irradiation at PSI (Figure 2) were opened in the hot cell using a cutting system, developed in-house, and transferred into a Reacti-Vial™ (a small glass v-vial generally used for radiopharmaceutical labeling) for further processing. A separate system was designed and constructed to open the target received from the Bern cyclotron laboratory. A representative picture of the latter opening setup is shown in Figure 3.

**Figure 4:**
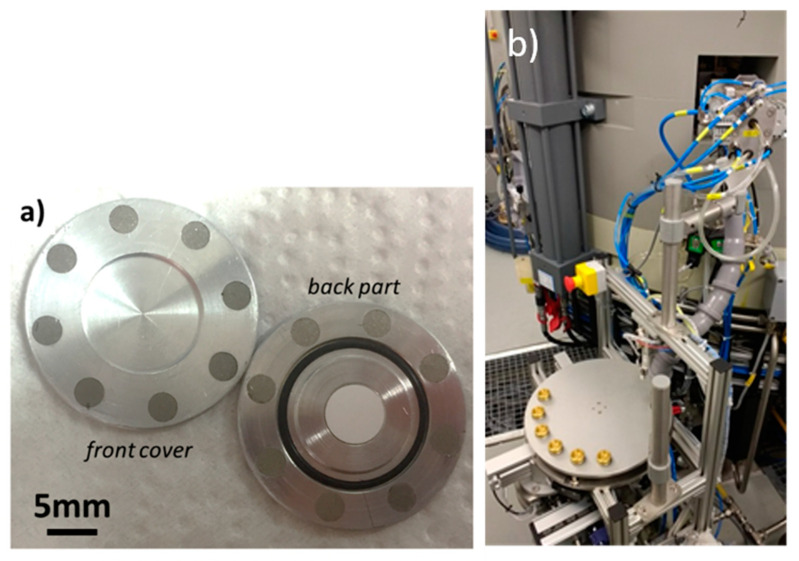
(**a**) Target coin containing a CaO pellet for irradiations using the solid target station at the Bern cyclotron laboratory. The front cover (lid) has a tunable beam entrance window, while the back part (cup) contains the pellet and the O-ring. Once put together, it forms a disc 24 mm in diameter and 2 mm thick. (**b**) The IBA Nirta solid target station and the solid target transfer system (STTS) installed on the Bern medical cyclotron.

Several irradiations were performed at the Bern medical cyclotron to demonstrate the feasibility of implementation of ^44^Sc production with such a cyclotron. One of the enriched ^44^CaO targets prepared for irradiation in Bern is shown in Figure 4a, where the 6 mm diameter pellet, the disc-shaped target coin sealed with permanent magnets and the rubber O-ring are visible. The advantage of using CaO pellets over CaCO_3_ was telling, as the CaCO_3_ target degassed and set off radiation protection alarms in the irradiation vault. The degassing from CaO targets, on the other hand, was modest and could be kept under control. The solid target station installed on the cyclotron and the pneumatic solid target transfer system are shown in Figure 4b. The station receiving the shuttle was equipped with a cadmium zinc telluride (CZT) detector to assess the activity produced at end of bombardment (EOB) [15,24]. This system was installed approximately 50 cm from the delivery point.

The irradiated targets were then transported from Bern to PSI (112 km by road; 2 h in duration). The coin target was introduced into the hot cell and fixed in place, in a slightly tilted position, by means of a suction tube. The front cover of the coin was removed with the use of the movable suction cup at the end of the tube. The encapsulated pellet fell out and through a funnel into a Reacti-Vial™ for further processing (Figure 3).

At PSI’s IP2 target station, the 72 MeV proton beam was degraded using niobium (Nb) at various thicknesses to provide the desired energy to irradiate the target in question. The target is cooled with water between the degrader and the target itself [23]. The size of degraders ranged from 1.0 to 3.5 mm and the degradation of the beam, calculated using SRIM-2013 [25]. The values stated are an approximation; however, as this does not take the beam (and energy) spread, as a result of the degradation of the proton beam, into consideration. When using a 3.4 mm Nb degrader, as for ^44^Sc production, the proton beam energy was calculated at ~10.3 MeV [23]. The targets proved to be robust in the proton beam for a period of 90 min at 40–50 µA using PSI’s IP2 irradiation station, as well as for a 5.45 h irradiation period at 18 µA at the IBA Cyclone 18 medical cyclotron at UniBe.

**Figure 5:**
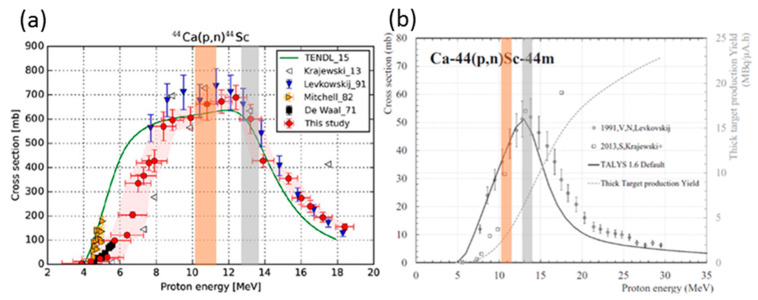
Excitation functions of (**a**) ^44^Sc (adapted from [21]: reprinted with permission from Elsevier) and (**b**) ^44m^Sc (adapted from [26]: reprinted with permission from IOP Publishing) using the ^44^Ca(p,n) nuclear reaction. The orange shade (PSI) and gray shade (UniBe) indicates the irradiation energy window of each facility, respectively.

While the yield of ^44^Sc between the energies used at PSI and UniBe were relatively similar due to the flatness of the cross section (Figure 5, left) [21], a difference in the production of ^44m^Sc (T_1/2_ = 58.6 h) was expected (Figure 5, right) [26]. Taking these two excitation function graphs into consideration, irradiating ^44^Ca targets at ~10.3 MeV was advantageous to that at ~13 MeV: the yield of ^44^Sc was marginally higher, while the production of ^44m^Sc side product decreased. At the Bern medical cyclotron, cooling of the target occurs with helium on the front part of the target and with water on the rear. The helium cooling is separated from the vacuum by means of an exit window, which is adjustable. A 500 µm-thick Al exit window was used for the experiments presented in this paper. The degrading issue can be addressed by either adjusting the exit window thickness, or optimizing the design of the magnetic coin by increasing the thickness of the aluminum entrance lid (thereby, degrading the beam energy) and decreasing that of the cup in order to maintain the same volume for the pocket hosting the pellet.

At the Bern medical cyclotron, the position of the beam was optimized to ensure that the maximum protons hit the 6 mm diameter target. This was done on the basis of two parameters: the beam current hitting the target coin and passing through the 12 mm aperture of a collimator, and the beam current collected by the collimator itself. The beam has about 10 mm full width half maximum (FWHM) and the position of the beam was varied by changing the position of the stripper in order to have the maximum protons on target. The beam current was then slowly increased, at a pace of 0.5 µA every 30 s, until the desired beam current (about 10 µA) was reached. The same procedure was followed when lowering the beam current at EOB. This procedure is fundamental to avoid thermal stress to the target and to monitor possible radioactive degassing.

### 2.3. Chemical Separation

Irradiation conditions, along with the yield of product, and its quality by means of molar activity determination are briefly described in Table 1 and Table 2 for PSI and UniBe, respectively. It must be noted that the irradiation of Target 1 at UniBe yielded vast activity (Table 2), where over 3.2 GBq ^44^Sc was obtained—9 h after EOB. The activity at EOB was estimated to be about 15 GBq obtained with 18.4 µA on the target disk (corresponding to ~5 µA on pellet) for 5.75 h.

**Figure 6:**
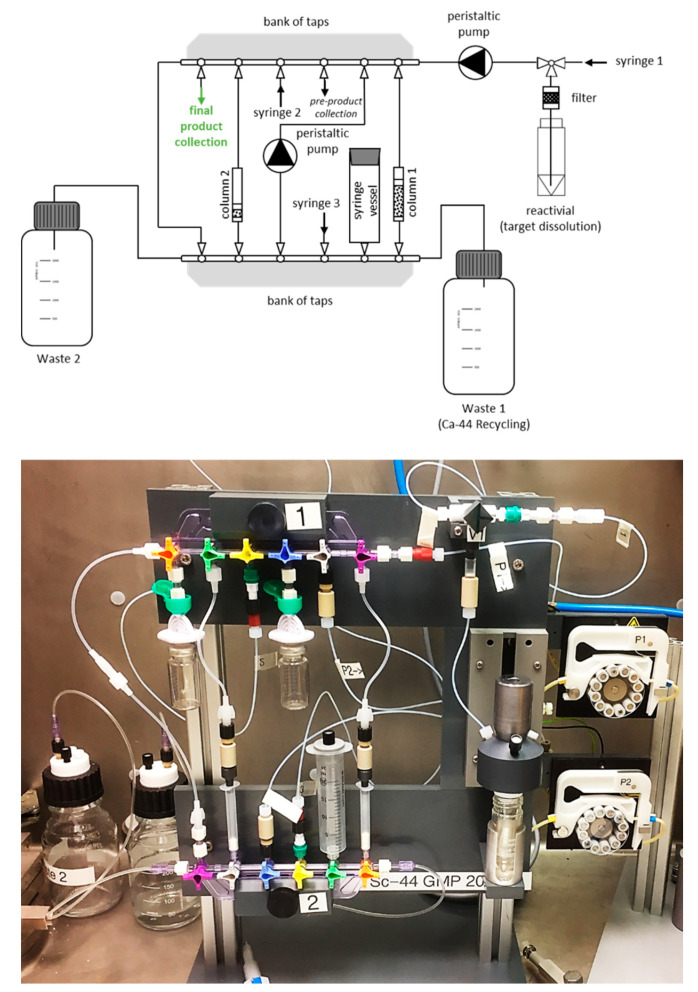
Schematic representation of the ^44^Sc chemical separation system (above), with the actual system—using disposable medical grade components—shown below. S1, S2, S3 in the scheme indicate the outside syringe lines (containing Bbraun non-return valves) to introduce the solutions used to perform the separation.

The targets were chemically processed (Figure 6) using a slightly modified separation system than before. While the system can be used as described by van der Meulen et al. (2015) [8], further developments were made to adapt it to the dissolution conditions of the new target design, as well as to cater for a preclinical product, as described in Domnanich et al. (2016) [13]. Irradiated targets, from PSI or UniBe, were easily dissolved in HNO_3_ (1.0 M, 3 mL) in order to perform the chemical separation, instead of using HCl as before. The use of HNO_3_ would ensure the elution and, thereby, separation of Fe(III) from the desired Sc(III) product [27], thereby, immediately eliminating an “environmental” contaminant (the use of “environmental” indicates that the contaminant was not initially part of the chemical separation system and may have been introduced from a laboratory environment, e.g., components used when pressing targets, rust from within the hot cell, etc.). The DGA extraction resin column was then rinsed with 10 mL 0.1 M HNO_3_, followed by 15 mL 3.0 M HCl in order to convert the column into chloride form and to get rid of any further impurities, respectively. ^44^Sc was eluted from the DGA column with 4 mL 0.1 M HCl.

When the product was to be used for preclinical application, a second DGA column was employed to enable elution of the activity in a small volume (700 μL) of 0.05 M HCl, which was used directly for labeling reactions. Where the product was envisaged for use in a clinical setting, the second column was replaced by a cartridge containing SCX cation exchange resin. In the latter case, the eluted solution had a high osmolarity and, as a result, the final radiopharmaceutical had to be purified via a cartridge as commonly performed for clinically employed radiopharmaceuticals.

A new chemical separation system was developed to adapt the production of ^44^Sc toward a GMP environment (Figure 6). The main part of the setup consists of individually packed, medical grade components (Vygon Schweiz GmbH) which were exchanged for every production run. This closed system minimizes the contamination of the final product with environmental, non-radioactive metal ions. This device was thoroughly tested toward producing ^44^Sc.

**Figure 7:**
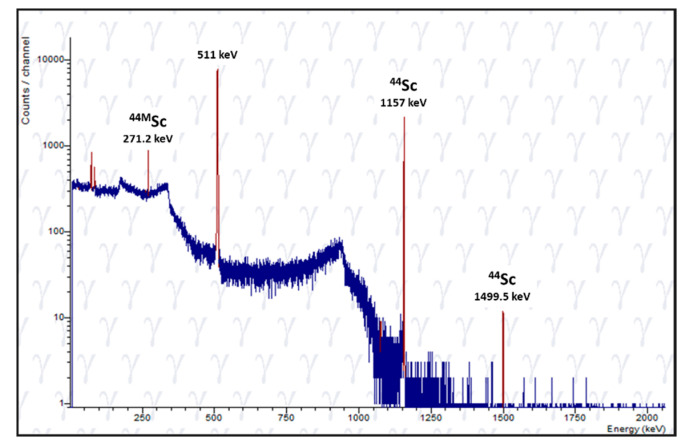
Representative γ-spectrum of ^44^Sc eluate from irradiated Ca targets, indicating the product is radionuclidically pure.

The developed target and separation method yielded 0.2–4.3 GBq ^44^Sc at the end of separation (EOS). Yields from selected production runs performed at PSI and UniBe are summarized in Table 1 and Table 2, respectively. The final two irradiations were proofs-of-principle to demonstrate that short irradiations were possible; however, this is not a viable option should one wish to ship the target. It could be processed onsite, however. Radionuclidic purity at EOS was determined using γ-spectrometry (Figure 7). The eluted product from targets irradiated at PSI contained ~0.5% ^44m^Sc, whereas the irradiation at UniBe resulted in ~2% of ^44m^Sc in the final product. This larger fraction of ^44m^Sc is partially explained by the longer time after EOB for the processing of UniBe targets. Furthermore, the product obtained from the productions contained trace activities of ^88^Y (T_1/2_ = 106.62 d). This is likely due to the purity of the enriched target material, where it contained traces of Sr (Supplementary Materials, Figures S10 and S11). As the Sr is irradiated, it produces ^88^Y via the ^nat^Sr(p,n)^88^Y nuclear reaction. ^88^Y discovered in the final product is similar to what was discovered when manufacturing ^43^Sc from enriched ^43^Ca target material [28]. The experimental production runs indicated an impurity of ~0.001% Y in the final product.

Fe, Zn, and Ni are regarded as potential environmental contaminants and it is important to ensure that they are eliminated from the final product. The chemical separation method was adjusted to ensure that these impurities are removed, should they have been introduced into the system along with the target material, as these elements are important competitors for DOTA chelation [16].

The quality of the ^44^Sc eluate with regard to its labeling efficiency was determined by testing the ability of radiolabeling using DOTANOC. HPLC analysis demonstrated a peak of free ^44^Sc at a retention time (*t_R_*) of 2.3 ± 0.1 min, while the *t_R_* of the radiopeptide was 9.4 ± 0.1 min. Molar activities of up to 25 MBq/nmol with labeling efficiencies of 99% were achieved. DOTANOC was labeled with ^44^Sc at 10 MBq/nmol at >99% radiochemical purity (irradiated at UniBe), while molar activity of 25 MBq/nmol was achieved with targets irradiated at PSI, presumably due to the larger amount of activity obtained at EOS and the possibility of immediate processing of the target.

It was subsequently found that the reading for the beam current on target and on the collimator at UniBe were not correct for some of the irradiations, due to an issue with electrical contacts (irradiation 2 to 5 in Table 2). This led to non-optimal beam centering. In some cases, only the tail of the beam was hitting the pellet, resulting in a low produced activity and overall low yields. This clearly underlines the importance of a correct beam positioning for the irradiation process. To evaluate where the beam struck the target, the front cover of the UniBe target coin was placed onto Gafchromic foils (Gafchromic EBT^2^, QD+, International Specialty Products, Wayne, NJ, USA) (Supplementary Materials, Figure S7). Other issues were encountered practically, when there were problems removing the target from the target holder. This was possibly due to the cooling of the target not being as efficient as desired. The target was either subsequently removed physically in the hot cell with a screwdriver (Run 3), or the target holder directly placed in the acid media and the target dissolved (Runs 4 and 5). Unfortunately, the magnets (as part of the target holder for the sealing mechanism) contained Sm and these runs resulted in poor radiolabeling capability. It was surmised that it could be due to the presence of Sm in the final product. Sm has similar chemical characteristics as Sc in the system using DGA resin and, as a result, the two elements could not be separated. This conclusion was substantiated by the literature [27], as well as subsequent mass spectrometry determination of the final product (Supplementary Materials, Figure S9).

These issues underline the importance of careful preparation of the target. In particular, the mass was crucial for the targets irradiated at the Bern medical cyclotron. If the mass was slightly lower than the nominal one, poor mechanical contact with the aluminum of the coin resulted in poor cooling. This leads to a large increase of temperature during irradiation, producing degassing, deformation and melting of the target material that then sticks to the coin and becomes difficult to remove. It was proven that the procedure of irradiation and target removal was smooth only for targets containing the nominal mass of material.

The enriched calcium collected in the segregated waste bottle from the first column was subsequently evaporated to dryness, picked up in dilute HCl before the addition of ammonium oxalate, as reported previously [8]; however, the final step involved heating to 900 °C to ensure conversion to CaO. It was shown that recycled material could be used effectively for ^44^Sc production, obtaining high yield and good quality product (Table 1). This is of paramount importance in view of commercial production.

Logistics with regard to irradiation timing and transport of targets are critical aspects in production arrangement. In particular, if a medical cyclotron is also used for other routine productions (typically ^18^F, as in the case of the Bern facility), schedule conflicts may happen and careful planning is required. Furthermore, the CaO target needs to be placed inside the target station and sealed with the piston as quickly as possible before irradiation to avoid its degradation. If a manual operation is needed, this may collide with high radiation levels in the cyclotron bunker due to other production activities.

Projects are ongoing to improve the full procedure at PSI and UniBe, in particular, to improve and standardize the preparation of the target. Importantly, the possibility to use Nb instead of Al for the construction of the magnetic coin is under investigation. Although more difficult to manufacture, Nb coins would be reusable and will almost not be activated during irradiation. Since the centering of the beam on the pellet is a critical issue, the development of a novel active irradiation system is ongoing at the Bern medical cyclotron. By means of a very compact focusing and steering magnet, this system will focus the beam on the 6 mm diameter pellet and keep it in position by means of an online feedback system, which analyzes the signals provided by a beam-monitoring detector [15]. These developments will likely improve the production yield and minimize unwanted radiation protection issues, as the activation of the coin outside the pellet area. Furthermore, the possibility to insert the target coin without entering the cyclotron bunker is under investigation, to make solid target irradiations as independent as possible from other production activities.

### 2.4. Preclinical Application of ^44^Sc-PSMA-ALB-02 Imaging

As a proof-of-concept study, the ^44^Sc produced at the medical cyclotron was used for a preclinical study using a previously developed albumin-binding PSMA ligand referred to as PSMA-ALB-02 [29]. ^44^Sc-PSMA-ALB-02 was readily prepared at a molar activity of 5 MBq/nmol with a radiochemical purity of >98% (Supplementary Materials, Figure S13).

**Figure 8:**
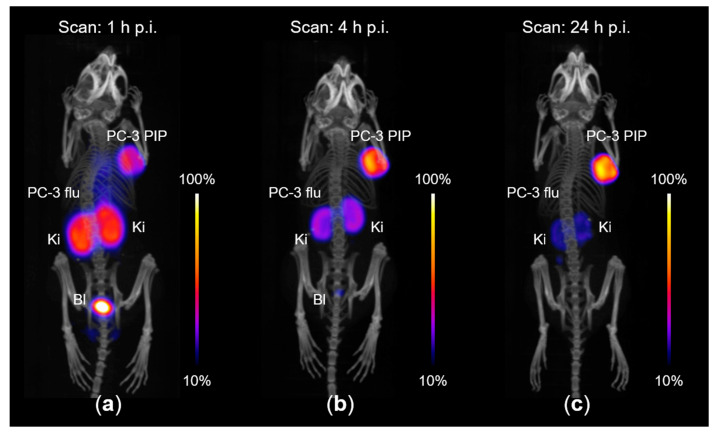
PET/CT images acquired with PC-3 PIP/flu tumor-bearing nude mice after intravenous injection of [^44^Sc]Sc-PSMA-ALB-02 (~5 MBq, 1 nmol per mouse). The PET/CT images are shown as maximum intensity projections (MIPs) with the scale adapted for each time point to make tumors and kidneys visible. (**a**) PET/CT scan obtained 1 h p.i. of the radioligand; (**b**) PET/CT scan obtained 4 h p.i. of the radioligand; (**c**) PET/CT scan obtained 24 h p.i. of the radioligand. PC-3 PIP = PSMA-positive tumor xenograft on the right shoulder; PC-3 flu = PSMA-negative tumor xenograft on the left shoulder; Ki = kidney; Bl = bladder.

PET/CT imaging studies were performed with PC-3 PIP/flu tumor-bearing mice 1 h, 4 h and 24 h after injection of ^44^Sc-PSMA-ALB-02 (Figure 8). PC-3 PIP tumor xenografts, located on the right shoulder, showed high uptake of activity, while no signal was detected in PC-3 flu tumors on the left shoulder. These findings confirmed the PSMA-specific tumor accumulation of ^44^Sc-PSMA-ALB-02. The PET/CT images were in agreement with post mortem data of the tumor uptake and distribution profile in other organs and tissues. Importantly, scanning at late time points was feasible even when less than 5% of injected activity was measured in the body of the mouse at 24 h p.i. (Figure 8c).

Quantitative results of the biodistribution of ^44^Sc-PSMA-ALB-02 are given in the Supplementary Materials (Figure S13).

The radiolabeling of PSMA-ALB-02 was readily achieved with ^44^Sc at high radiochemical purity (>98%), comparable to the results previously obtained for ^44^Sc-PSMA-617 [10]. Preclinical PET/CT experiments demonstrated the option of using the ^44^Sc-PSMA-ALB-56 for visualization of PSMA-positive tumors, even at late time points after injection, which may be of interest for future clinical translation of this promising PET radionuclide.

## 3. Materials and Methods

Natural calcium carbonate (CaCO_3_, ≥99%, anhydrous; Sigma Aldrich GmbH, Steinheim, Germany), was used for test targets. Targets for ^44^Sc production were prepared with enriched ^44^Ca calcium carbonate (^44^CaCO_3_, 97.00% enriched; TRACE Sciences International, Wilmington, DE, USA). Chemical isolation was performed with *N*,*N*,*N*’,*N*’-tetra-n-octyldiglycolamide, non-branched resin (DGA extraction chromatographic resin, 50–100 µm; Triskem International, Bruz, France), SCX cartridges (bond elute SCX cartridge, 100 mg, 1 mL, 40 µm; BGB Analytik AG, Boeckten Switzerland), nitric acid (HNO_3_, 65%, Suprapur; Merck KGaA, Darmstadt, Germany), hydrochloric acid (HCl, 30%, Suprapur; Merck KGaA, Darmstadt, Germany), sodium chloride (NaCl; Sigma Aldrich GmbH, Steinheim, Germany) and Milli-Q water. DOTANOC (ABX GmbH, Radeberg Germany) was used for the radiolabeling of the product to determine radiochemical purity, with sodium acetate (Alfa Aesar, Kandel, Germany; 0.5 M, pH 8) to adjust the pH. Other chemicals used in the quality control process included trifluoroacetic acid (Sigma Aldrich GmbH, Steinheim, Germany) and acetonitrile (HPLC Grade; VWR Chemicals, Radnor, PA, USA). All chemicals were used as received, without further purification.

### 3.1. Target Preparation

Enriched calcium oxide (^44^CaO) was obtained via thermal decomposition of ^44^CaCO_3_ at 900 °C for 1 h under Ar-flow. The resultant CaO powder (~30 mg) was pressed into disc-shaped pellets, 6 mm in diameter and 0.5 mm thick, by applying an axial pressure of 2 tons for 5 s. The pellet was either immediately encapsulated in aluminum, stored under Ar atmosphere or in a desiccator under vacuum until further use, as the material can absorb moisture from the air. X-ray diffraction (XRD;) measurements of the target material were performed using a Bruker D8 Advance powder diffractometer (Bruker Corporation, Fällanden, Switzerland) equipped with a M. Braun 50 m position sensitive detector, Bragg-Brentano geometry, Cu Kalpha1 radiation (1.54059 Å), focusing Ge-monochromator.

### 3.2. Target Irradiation

The resulting target was irradiated for 90 min using the 72 MeV proton beam (at 50 µA), provided by Injector 2 at PSI. The beam was degraded to ~10.3 MeV using a Nb disc (3.4 mm) [23], to optimize and produce high yields of ^44^Sc by using the ^44^Ca(p,n)^44^Sc nuclear reaction. Similar targets were tested using different beam currents (40–65 µA) and irradiation times (0.5–1.5 h). Irradiated targets were transferred into a hot cell for further processing.

The ^44^Ca(p,n)^44^Sc nuclear reaction was used to implement ^44^Sc production methods at the Bern cyclotron laboratory [30]. This facility is equipped with an IBA Cyclone HC cyclotron providing 18 MeV proton beams with currents up to 150 µA. Proton beams are obtained by stripping H^−^ accelerated ions in single or dual beam mode. This accelerator is routinely used for the production of ^18^F-labeled PET radiotracers, as well as multi-disciplinary research activities by means of a dedicated 6-m-long Beam Transfer Line (BTL), ending in a second bunker with independent access. The BTL was used to measure the cross section of the ^44^Ca(p,n)^44^Sc nuclear reaction [21].

A specially designed magnetic “coin” was used for the irradiation, encapsulating the 6-mm diameter 500-µm thick ^44^CaO pellets [15,24]. This device was constructed using an aluminum alloy (EN AW-6082), composed of two parts, forming a 24-mm diameter 1-mm thick disc. The two parts were held together by means of permanent magnets. The front window of the coin was used to adjust the energy of the protons reaching the target material. A good thermal contact between the two halves was assured to optimize cooling, while an O-ring was present to minimize radioactive gas release during irradiation. This special target was fully compatible with the IBA Nirta solid target station installed in one of the out-ports of the cyclotron, together with a pneumatic solid target transfer system (STTS) by TEMA Sinergie, Italy. The coin was manually inserted in the target station, which was customized in such a way that the shuttle containing the irradiated target could be sent to a hot cell in the nearby GMP radiopharmacy, or to a reception station in the BTL bunker. The latter was used to collect the irradiated targets that were transported to PSI for chemically processing.

### 3.3. Chemical Separation

The chemical separation to isolate ^44^Sc from impurities and its target material was modified slightly from the previously-reported methods [8,13]. In brief, the target was dissolved in 3 mL 1.0 M HNO_3_ and loaded onto a column containing DGA extraction resin (87 mg DGA, preconditioned with 3 mL 1.0 M HNO_3_). The rinsing of the target container and the DGA column with 1.0 M HNO_3_ ensured a complete transfer of the ^44^Sc radioactivity and complete removal of residual Ca, respectively. The column was then rinsed with 10 mL 0.1 M HNO_3_, followed by 15 mL 3.0 M HCl in order to convert the column into chloride form and to get rid of impurities, respectively. ^44^Sc was eluted from the DGA column with 4 mL 0.1 M HCl. Subsequently, the solution was acidified with the addition of 4 mL 6.0 M HCl to yield a 3.0 M HCl solution, which was then passed through a second DGA column (43 mg DGA, preconditioned with 3 mL HCl), to which the ^44^Sc activity was sorbed. The elution of ^44^Sc from the second column was performed with 700 μL 0.05 M HCl and used directly for radiolabeling toward preclinical studies. When the product was not used for preclinical applications, the second column was replaced with a SCX cartridge, the elution of column 1 in 0.1 M HCl directly loaded onto the SCX column (without acidification) and ^44^Sc eluted therefrom using 700 μL of a 4.85 M NaCl/0.13 M HCl mixture. The ^44^Ca containing waste from the first DGA column was collected and processed as previously described [8] in order to recover the target material. The radionuclidic purity of the ^44^Sc eluate was investigated by γ-spectrometry using an N-type high-purity germanium (HPGe) coaxial detector (EURISYS MEASURES, Montigny-Le-Bretonneux, France) and the Ortec InterWinner version 7.1 software (Ortec, Atlanta, GA, USA).

### 3.4. Radiolabeling

The total ^44^Sc activity in the eluate obtained after separation was quantitatively determined using a dose calibrator (ISOMED 2010, Nuclear-Medizintechnik Dresden GmbH, Dresden, Germany). A ~80 MBq aliquot was removed from the eluate for quality control purposes. The pH was adjusted to 4.5 with the addition of sodium acetate solution (0.5 M, pH 8) and an aliquot from DOTANOC stock solution (1 mM, up to 70 μL, 0.7 nmol/μL) was added in order to obtain a molar activity between 5–50 MBq/nmol. The reaction mixture was incubated at 95 °C for 15 min. High-performance liquid chromatography (HPLC) with a C-18 reversed-phase column (Xterra^TM^ MS, C18, 5 µm, 150 × 4.6 mm; Waters) was used for quality control. The mobile phase consisted of water with 0.1% trifluoracetic acid (A) and acetonitrile (B) with a gradient of 95% A and 5% B to 20% A and 80% B over a period of 15 min at a flow rate of 1.0 mL/min.

Alternatively, the radiolabeling capability of ^44^Sc product at different DOTA-to-nuclide molar ratios was assessed by thin layer chromatography (TLC). Several aliquots of ~1 MBq ^44^Sc were removed from the eluate and added to prepared DOTA dilutions in 0.5 M sodium acetate (pH 4.5). The reaction mixtures were incubated for 30 min at 95 °C and 2 µL of each solution deposited on TLC silica gel 60 F_254_ plates, used as stationary phase. A mixture of ammonium acetate (10% *w*/*v* aqueous solution) with methanol (1:1 *v*/*v*; pH 5.5) was used as mobile phase. After completion of the chromatographic separation, the TLC plate was used to expose a phosphor screen (Multisensitive, Perkin Elmer Inc., Boston, MA, USA) followed by reading using a Cyclon Phosphor Imager (Perkin Elmer Inc., Boston, MA, USA). Peaks corresponding to uncoordinated ^44^Sc and ^44^Sc-DOTA were integrated using the OptiQuant image analysis software (version 5.0, Perkin Elmer Inc., Boston, MA, USA).

### 3.5. Preclinical Imaging

PSMA-ALB-02 consisting of the glutamate-urea-lysine-based PSMA-binding entity, an albumin-binding entity to enhance blood circulation and a DOTA chelator for coordination of the radiometal was previously developed at PSI [29] (Supplementary Material, Figure S12).

### 3.6. Preparation of ^44^Sc-PSMA-ALB-02

PSMA-ALB-02 was labeled with ^44^Sc, as previously reported for PSMA-617 [10]. In brief, a stock solution of PSMA-ALB-02 (1 mM) was mixed with a solution of ^44^Sc, adjusted to pH 3.5–4.0 with sodium acetate solution (0.5 M, pH 8). The reaction mixture was incubated for 15 min at 95 °C. Quality control of ^44^Sc-PSMA-ALB-02 was performed using HPLC, as reported above.

### 3.7. Tumor Mouse Model

All applicable international, national, and/or institutional guidelines for the care and use of animals were followed. In particular, all animal experiments were carried out according to the guidelines of Swiss Regulations for Animal Welfare. The preclinical studies were ethically approved by the Cantonal Committee of Animal Experimentation and permitted by the responsible cantonal authorities (license number 75668).

PC-3 PIP and PC-3 flu tumor cells were kindly provided by Prof. Dr. Martin Pomper (Johns Hopkins University School of Medicine, Baltimore, MD, USA) [31]. Before inoculation, the cells were grown in RPMI cell culture medium supplemented with 10% fetal calf serum, L-glutamine and antibiotics, as well as puromycin (2 µg/mL), to maintain PSMA expression [32].

Female, athymic BALB/c nude mice were obtained from Charles River Laboratories (Sulzfeld, Germany) at the age of 5–6 weeks. They were subcutaneously inoculated with PC-3 PIP cells (6 × 10^6^ cells in 100 μL Hank’s balanced salt solution (HBSS)) on the right shoulder and with PC-3 flu cells (5 × 10^6^ cells in 100 μL HBSS) on the left shoulder. Biodistribution and PET imaging studies were performed approximately 2 weeks after PC-3 PIP/flu tumor cell inoculation.

### 3.8. Imaging Studies

PET/CT scans were performed using a small-animal bench-top PET/CT scanner (G8, Perkin Elmer, Boston, MA, USA), as previously reported [10]. During the scans, the mice were anesthetized with a mixture of isoflurane and oxygen. Static whole-body PET scans of 10 min duration were performed at 1 h, 4 h, and 24 h after injection of ^44^Sc-PSMA-ALB-02 (~5 MBq, 1 nmol) followed by a CT scan of 1.5 min. All images were prepared using *VivoQuant* post-processing software (version 3.5, inviCRO Imaging Services and Software, Boston, MA, USA). The images presented with the scale adjusted for each time point (3-fold increased for the 4 h time point and 30-fold increased for the 24 h time point relative to the 1-h time point scale) to allow visualization of the most important organs and tissues.

## 4. Conclusions

The work reported in this article demonstrated that enriched ^44^Ca targets can be irradiated successfully using a medical cyclotron, equipped with a commercial solid target station, to obtain ^44^Sc with high yields and radiochemical purity. Radioactive degassing during irradiation from the target decreased using the oxide over carbonate material. Preclinical imaging using ^44^Sc-PSMA-ALB-02, from the activity produced at the medical cyclotron, was successfully performed. The promising results reported herein can be considered to be the basis towards the implementation of routine ^44^Sc production in view of theragnostic applications in nuclear medicine.

## Figures and Tables

**Table 1:** Irradiation of a selection of ^44^CaO targets (6 mm × 0.5 mm; ~30 mg) using a degraded 72 MeV proton beam at PSI.

Target No.	Time (h)	Beam Current (µA)	Integral (µAh)	Yield (EOS, GBq)	Quality Control with DOTANOC (MBq/nmol) †
1	1.67	45	75	3.81 *	25 [~99%]
2	1.5	50	75	2.50	13 [~98%]
3	1.5	50	75	4.28	25 [~99%]
4	1.5	50	75	3.97	25 [~99%]

* recycled target material used. † single measurement performed.

**Table 2:** Irradiation of ^44^CaO targets (6 mm × 0.5 mm, ~30 mg) using 18 MeV protons at the Bern medical cyclotron.

Target No.	Time (h)	Beam Current (µA) *	Integral (µAh)	Yield (End of Separation, GBq)	TLC Quality Control with DOTA (MBq/nmol) †
1	5.75	18.4	106	3.210	10 [~99%] **
2	4	5	20	0.704	17 [~93%]
3	4	10	40	0.144	1 [~97%] 5 [~70%]
4	4	10	40	0.197	1 [~58%]
5	4	7	28	0.467	3 [~60%]
6	5	7	35	0.580	22 [~96%] 14 [~99%]
7	0.5	5	2.5	0.2	N/A
8	0.5	7	3.5	<0.1	N/A

* Current reaching the target disc passing through a 12 mm-diameter collimator. The current hitting the 6 mm-diameter pellet located at the center of the disc is approximately 1/4, depending on the centering of the beam that has a quasi-gaussian shape. ** QC done with DOTANOC (HPLC) method. † single measurement performed.

## Supplementary Materials

The following are available online, Figure S1. X-ray diffraction patterns of CaO powder exposed to air at different time points. Figure S2. Profile overlap of front and back part of the IBA cyclotron target holder measured on a KEYENCE VR-3000 G2 profilometer. Figure S3. X-ray diffraction (intensity) patterns of CaO pellets encapsulated in a magnetic coin target holder and stored under air or under vacuum. Figure S4. Example of target that was left exposed to the elements for too long, resulting in the CaO target material absorbing moisture and resulting in rupture. Figure S5. Exhaust radioactivity in air of the Bern cyclotron bunker on the day of the first test with a CaCO_3_ target. Figure S6. Exhaust radioactivity in air of the whole Bern cyclotron laboratory during a typical irradiation of a CaO target for ^44^Sc production. Figure S7. Gafchromic foils after exposure to the irradiated front cover of the magnetic coin target holders used for ^44^Sc production at the Bern medical cyclotron. Figure S8. Schematic representation of a target holder placed in HNO_3_ in order to dissolve the sticking target out of the holder Figure S9. LC-ESI-TOF-MS spectra of DOTA-NOC reference sample, decayed ^44^Sc-DOTANOC sample of IBA cyclotron production run, theoretical Sm-DOTANOC (d). Figure S10. Long-term γ-spectrometry of partially decayed ^44^Sc eluate. Figure S11. Excerpt from Certificate of Analysis for enriched ^44^CaCO_3_ from TRACE Sciences, USA Figure S12. Chemical structure of PSMA-ALB-02 consisting of a DOTA-chelator, an albumin-binding entity and a glutamate-urea-lysine (Glu-urea-Lys)-based PSMA-binding entity. Figure S13. Representative chromatogram of ^44^Sc-PSMA-ALB-02, demonstrating the product peak and its retention time in minutes (t_R_ = 11.2 min). Figure S14. Biodistribution data obtained 1 h and 4 h after injection of [^44^Sc]Sc-PSMA-ALB-02 in PC-3 PIP/flu tumour-bearing nude mice. Table S1: LC-ESI-TOF-MS operating parameters.
