# Supplementary material for: Developments toward the Implementation of 44Sc Production at a Medical Cyclotron
Source: Molecules. 2020 Oct 14;25(20):4706. doi: 10.3390/molecules25204706 (PMC7587374; doi:10.3390/molecules25204706)
Supplement: Supplementary file 1 [file molecules-25-04706-s001.pdf]

molecules

Article: Supplementary Information

Developments towards the Implementation of ^{44}Sc Production at a Medical Cyclotron

Nicholas P. van der Meulen[1,2*], Roger Hasler[2], Zeynep Talip[2], Pascal V. Grundler[2], Chiara Favaretto[2], Christoph A. Umbricht[2], Cristina Müller[2], Gaia Dellepiane[3], Tommaso S. Carzaniga[3], Saverio Braccini[3]

[1] Laboratory of Radiochemistry, Paul Scherrer Institute, 5232 Villigen-PSI, Switzerland
[2] Center of Radiopharmaceutical Sciences ETH-PSI-USZ, Paul Scherrer Institute, 5232 Villigen-PSI, Switzerland
[3] Albert Einstein Center for Fundamental Physics, Laboratory of High Energy Physics, University of Bern, 3012 Bern, Switzerland
* Correspondence: nick.vandermeulen@psi.ch

Content

1. Challenges of CaO target preparation
2. Comparison of degassing of $CaCO_3$ and CaO targets during irradiation
3. Investigation of beam centering using gafchromic films
4. LC-ESI-TOF-MS analysis
5. Radionuclidic impurities
6. Radiolabeling and preclinical application of ^{44}Sc-PSMA-ALB-56
7. References

1. Challenges of CaO target preparation

Upon exposure to air, CaO undergoes hydration through adsorption of moisture, yielding calcium hydroxide and subsequent carbonation (which involves CO_2 fixation) to form calcium carbonate. The net reaction of this process can be represented by:

$$CaO \xrightarrow{+H_2O} Ca(OH)_2 \xrightarrow{+CO_2} CaCO_3 + H_2O \qquad \text{(Eq. 1)}$$

The hydration process is fast, while the following carbonation process is slow, due to the difference in water and CO_2 concentration in air [1]. This indicates that, when using CaO as target material, further measures have to be taken in order to prevent hydration and carbonation.

The severity of such measures was investigated by exposing prepared CaO powder to air and measuring X-ray Diffraction (XRD) spectra at different exposure times (0 h, 4 h, 16 h; Figure S1). Peaks corresponding to the diffraction pattern of $Ca(OH)_2$ can be identified in the spectra after 16 h exposure. The data, however, indicates that the overall crystallinity of the material decreases over time, as can be seen in the spectra recorded after 4 h. This could mean amorphisation of the present phases (CaO and $Ca(OH)_2$) and/or a formation of an amorphous $CaCO_3$ phase. Such secondary phase formations lead to changes of the crystal structure and result in volume changes (density $\rho_{CaO} > \rho_{CaCO3} > \rho_{Ca(OH)2}$). This has negative effects on prepared pellets from CaO powder, leading to cracking and breaking when exposed to air. Taking this into consideration, directly after conversion of $CaCO_3$ the resultant CaO powder was pressed into target pellets, while the exposure time was kept to a minimum (≤ 1 h). The resultant targets were encapsulated and stored under inert gas or in a desiccator under vacuum.

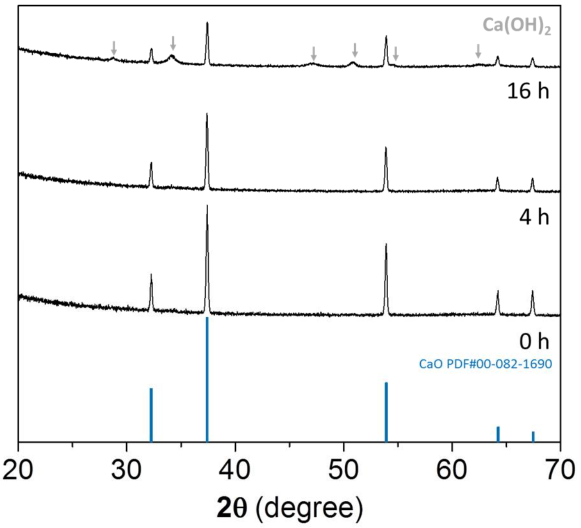

Figure S1. X-ray diffraction patterns of CaO powder exposed to air at different time points. Arrows indicate the $Ca(OH)_2$ phase.

Target holders used for irradiations at the Bern medical cyclotron only seal completely when pressed together with 6 bars of pressure within the target station. Outside the target station, held together only by the magnets of the "coin", the target holder exhibits a gap between front cover and back part, as confirmed by three-dimensional measurements using a KEYENCE VR-3000 G2 profilometer (Figure S2). It is important, therefore, to store such encapsulated targets under inert conditions to prevent secondary phase formations as indicated by XRD (Figure S3). Disastrous results were observed when such measures were not taken in the preparation of targets (Figure S4).

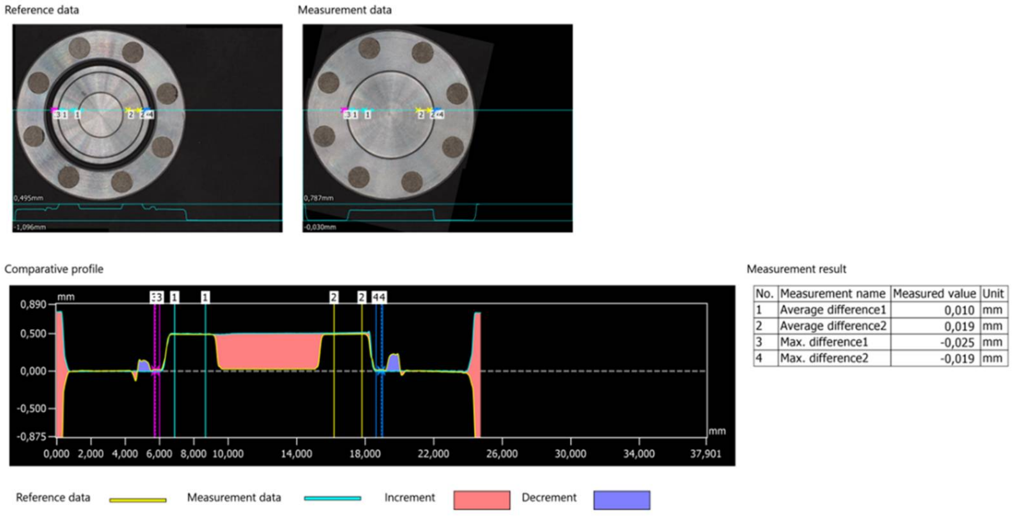

Figure S2. Profile overlap of front and back part of the IBA cyclotron target holder measured on a KEYENCE VR-3000 G2 profilometer. Comparison of the profiles indicates an insufficient sealing if just closed by magnets without external pressure.

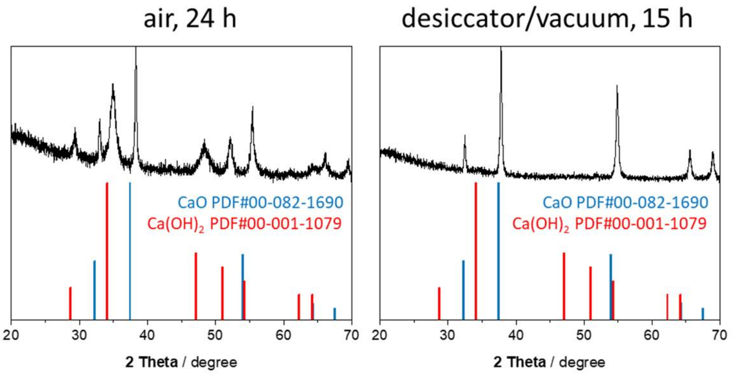

Figure S3. X-ray diffraction (intensity) patterns of CaO pellets encapsulated in a magnetic coin target holder and stored under air (*left*) or under vacuum (*right*). Storage of encapsulated targets in air leads to a crystalline $Ca(OH)_2$ phase formation and amorphisation of overall crystallinity (as indicated by an increased signal-to-noise ratio in the normalized spectra).

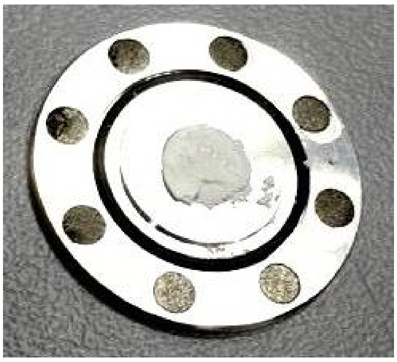

Figure S4. Example of target that was left exposed to the elements for too long, resulting in the CaO target material absorbing moisture and resulting in rupture. The target could not be used for irradiation.

2. Comparison of degassing of $CaCO_3$ and CaO targets during irradiation

The first irradiation was performed at the Bern medical cyclotron using $CaCO_3$ pellets. This irradiation had to be stopped after a few minutes, at beam currents of about 1 μA, due to an air contamination radiation safety alarm provoking the shutdown of the ventilation in the cyclotron bunker. Figure S5 shows the measurement of air contamination at the main exhaust of the facility on the day irradiation. The signals (shown as broad peaks), due to ^{41}Ar produced in air by neutrons via the ^{40}Ar(n,γ)^{41}Ar nuclear reaction during routine ^{18}F production, are clearly visible together with the huge peak due to the ^{44}Sc production test. The peak can be explained by the fact that, due to the increase of temperature, $CaCO_3$ decomposed to CaO and CO_2, leading to high pressure inside the capsule that decreased the thermal exchange between the pellet and the coin. This provoked the melting on the O-ring and the release of a large amount of radioactive gas. The analysis of the half-life of the exhaust gas showed that it consists of ^{13}N produced by the reaction ^{16}O(n, α)^{13}N [2]. This negative result demonstrated that the use of $CaCO_3$ pellets is not possible. For this reason, $CaCO_3$ pellets were abandoned and CaO targets developed.

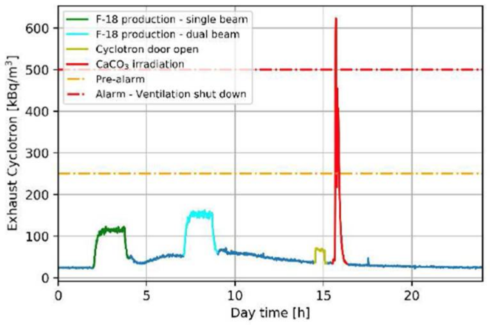

Figure S5. Exhaust radioactivity in air of the Bern cyclotron bunker on the day of the first test with a $CaCO_3$ target [2].

The use of CaO pellets proved to be successful also from a radiation protection point-of-view. The radioactive gas released by the target was found to be far from the alarm values and had little influence on the total amount of radioactivity released in the atmosphere by the whole facility, as shown in Figure S6.

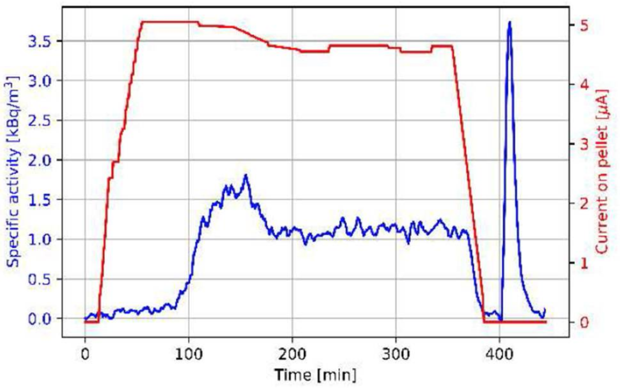

Figure S6. Exhaust radioactivity in air of the whole Bern cyclotron laboratory during a typical irradiation of a CaO target for ^{44}Sc production. The peak after EOB corresponds to the transfer of the irradiated target disc [2].

The amount of released radioactivity in form of ^{13}N was found to be comparable to ^{41}Ar produced during ^{18}F runs, thus, causing no problem from a radiation safety perspective. As shown in Figure S6, a peak was observed after EOB due to the release of radioactive gas as soon as the piston pressing the target in the solid target station was released. For this reason, a conservative waiting period of about 30 minutes after EOB (corresponding to about three ^{13}N half-lives) was adopted before releasing the piston to reduce the release of radioactive gas into the air of the bunker.

3. Investigation of beam centering using Gafchromic films

It was found that the reading of the beam current on target and on the 12 mm diameter collimator was not correct over the irradiation periods 2 to 5 (Table 2). This was due to a faulty connection leading to a bad centering of the beam on the pellet. In some cases, only the tail of the beam was hitting the target material, thus, producing low yields. In order to evaluate where the beam hit the target, the front cover of target coin was placed in contact with Gafchromic film (Gafchromic EBT2, QD+, International Specialty Products, U.S.A.) (Figure S7).

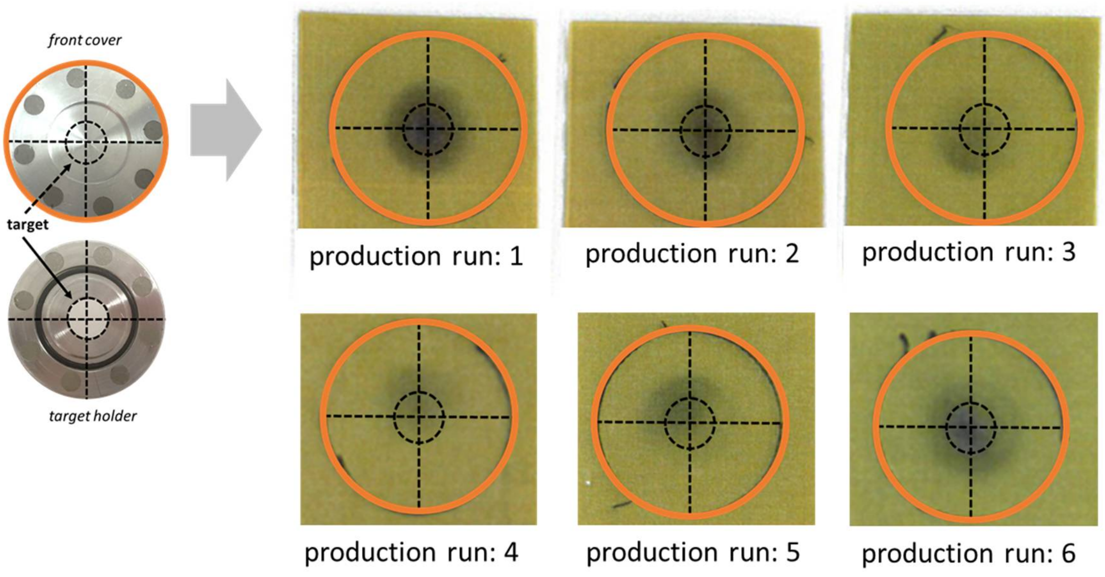

Figure S7. Gafchromic foils after exposure to the irradiated front cover of the magnetic coin target holders used for ^{44}Sc production at the Bern medical cyclotron. The dotted circle corresponds to the 6 mm diameter pellet.

It can be seen that the beam was not correctly centered for runs 3, 4 and 5. These experimental measurements demonstrate the importance of an accurate positioning of the beam.

4. LC-ESI-TOF-MS analysis

For some of the irradiations performed at the IBA cyclotron, problems were encountered in removing the irradiated target from the target holder. In these cases (production runs 3 – 5), the target holder was placed directly into the 1.0 M HNO_3 solution and the target dissolved. This led to a leaching of Sm from the samarium-cobalt magnets (part of the target holder sealing mechanism) (Figure S8) [3]. This resulted in a poor radiolabeling capability of the final product (see Table 2, main manuscript), since Sm and Sc show similar behavior on DGA resin under the given conditions and, therefore, cannot be separated. [4] Potential leaching of Co from the magnets does not contaminate the final product, because Co does not sorb on DGA resin in HNO_3 and, therefore, is washed out in the first step of the separation procedure. [4]

LC-ESI-TOF-MS analysis was performed on the decayed final product to confirm the presence of Sm in the eluate (Figure S9). To prepare the sample, an aliquot (~20 µL) of ^{44}Sc product solution was diluted to 25 µL using 0.05 M HCl and the pH adjusted to 4.5 with 0.5 M sodium acetate. DOTANOC peptide (2 µL, 1 mM) was added and the sample incubated at 95 °C for 15 min. A reference sample consisting of HCl, sodium acetate and DOTANOC was prepared using the same procedure. A 10-µL aliquot of the resultant sample, mixed with 20 µL EDTA solution, was used for LC-ESI-TOF-MS analysis using the operating parameters specified in Table S1.

Table S1. LC-ESI-TOF-MS operating parameters.

**LC conditions**	
Column ACE	ACE (150 * 3 mm), 3 µm C18
Flow rate	0.6 mL/min
Column temperature	40 °C
Injection volume	20 µL
Mobile phase	A: ACN 0.1% formic acid

	B: H_2O 0.1% formic acid

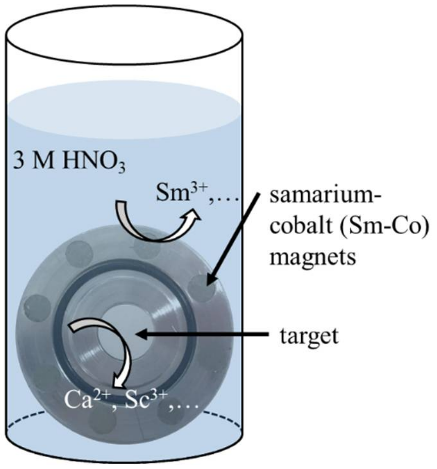

Figure S8. Schematic representation of a target holder placed in HNO_3 in order to dissolve the sticking target out of the holder. The strong acidic condition led to leaching of Sm and other contaminants from the Sm-Co magnets into the target solution.

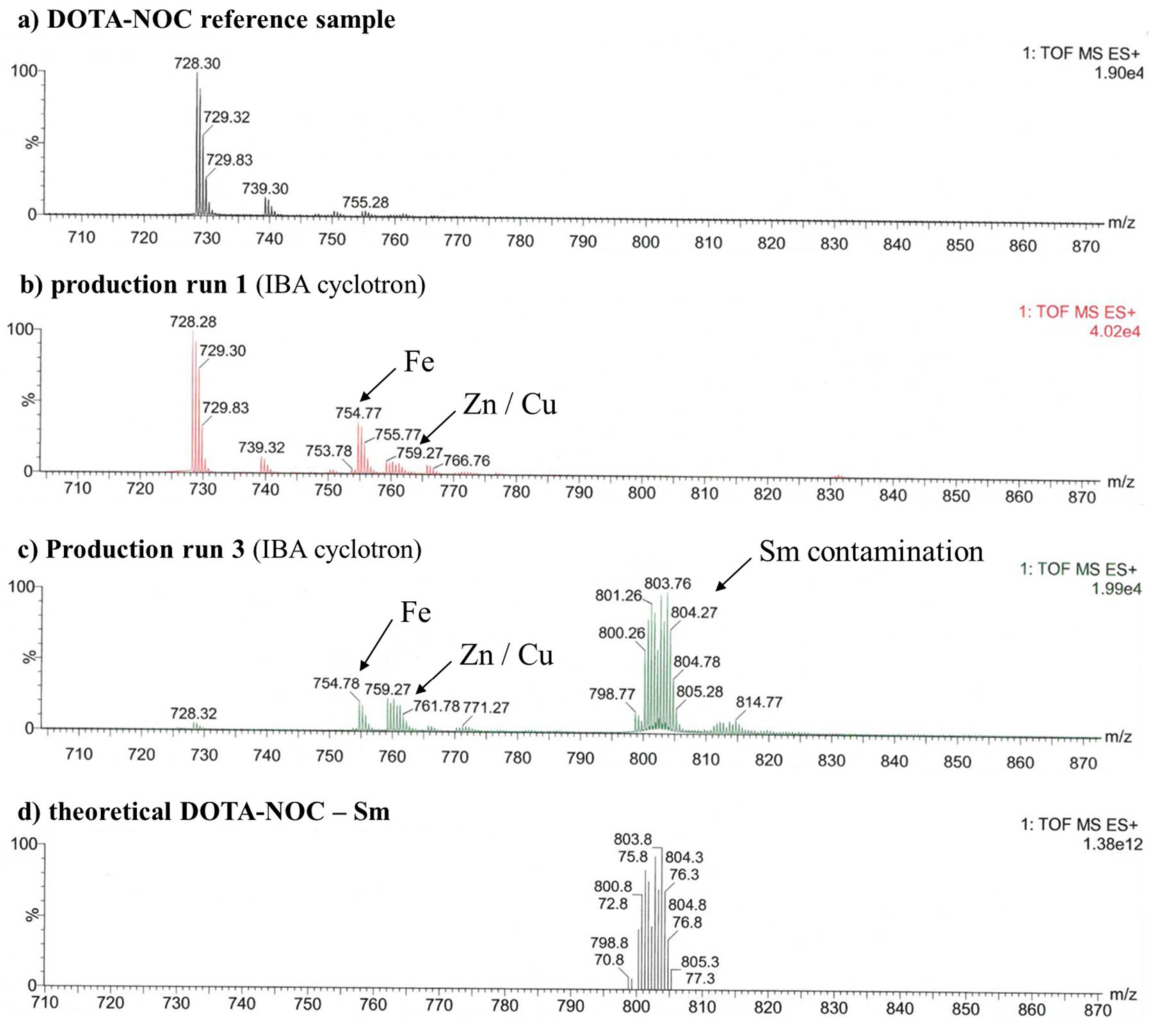

Figure S9. LC-ESI-TOF-MS spectra of DOTA-NOC reference sample (a), decayed ^{44}Sc-DOTANOC sample of IBA cyclotron production run 1 (b) and 3 (c), theoretical Sm-DOTANOC (d).

5. Radionuclidic impurities

In order to evaluate the amount of low-activity radionuclidic impurities, long-term γ-spectrometry of partially decayed ^{44}Sc eluate was measured (Fig. S10). Other than the final product containing ^{44}Sc and ^{44m}Sc (~2 %), trace amounts of ^{88}Y (~0.0013 %) were also discovered. This is likely due to the Sr impurity in the target material (Figure S11), where the ^{88}Y is formed via the ^{nat}Sr(p,n)^{88}Y nuclear reaction.

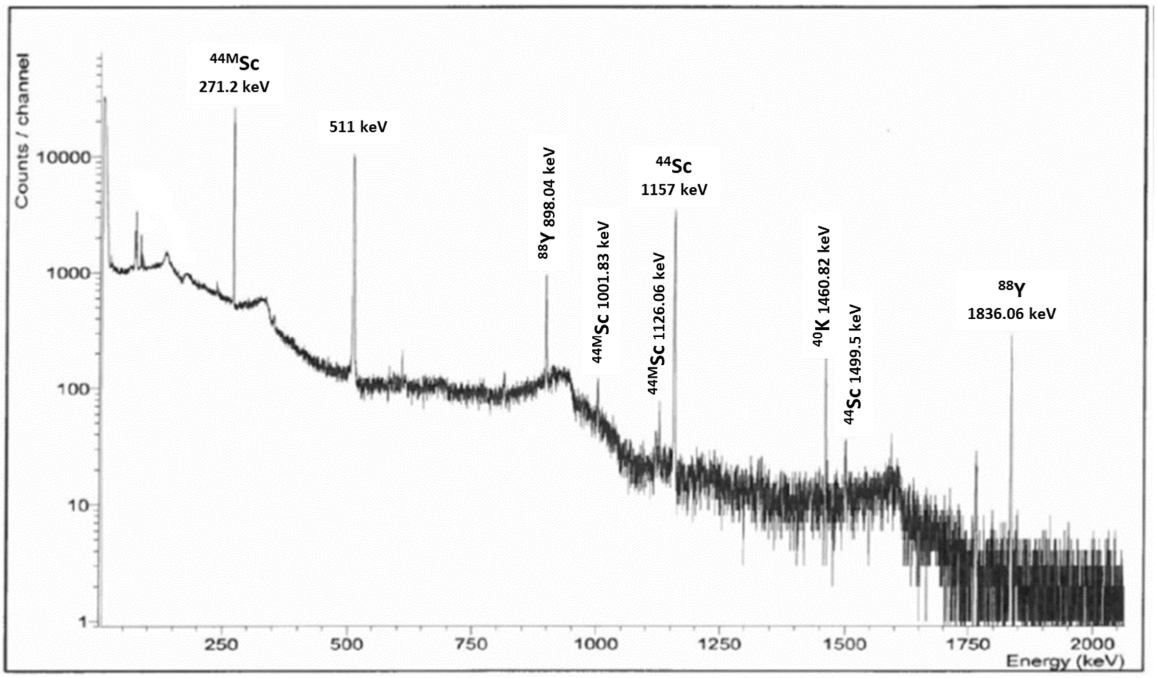

Figure S10. Representative long-term γ-spectrometry of partially decayed ^{44}Sc eluate (Dead time = 0.16%, count rate = 35.779 cps, Acquisition Time = 73731 s). Measurements were performed about 1 month after EOS.

Form: $CaCO_3$ (Carbonate)

Isotopic composition:

Isotope	40	42	43	44	46	48
Enrichment (%)	2.89	0.06	0.03	**97.00+/-0.2**	<0.002	0.02

Chemical Impurities:

Element	Symbol	Impurity Measurement (ppm)
Aluminum	Al	30
Barium	Ba	<20
Chlorine	Cl	20
Copper	Cu	30
Iron	Fe	40
Magnesium	Mg	70
Manganese	Mn	<10
Sodium	Na	<20
Nickel	Ni	<10
Lead	Pb	40
Silicon	Si	<30
Strontium	Sr	160

Figure S11. Excerpt from Certificate of Analysis for enriched $^{44}CaCO_3$ from TRACE Sciences, U.S.A.

6. Radiolabeling and preclinical application of ^{44}Sc-PSMA-ALB-56

6.1 Preparation of ^{44}Sc-PSMA-ALB-02

The chemical structure of PSMA-ALB-02 ligand is shown in Figure S12. It comprises a DOTA-chelator for coordination of the ^{44}Sc. The *p*-iodophenyl-moiety was used an albumin binder in order to increase the radioligand's blood circulation time and, hence, uptake in the tumor.

A representative HPLC chromatogram of the quality control of ^{44}Sc-PSMA-ALB-02 is shown in Figure S13.

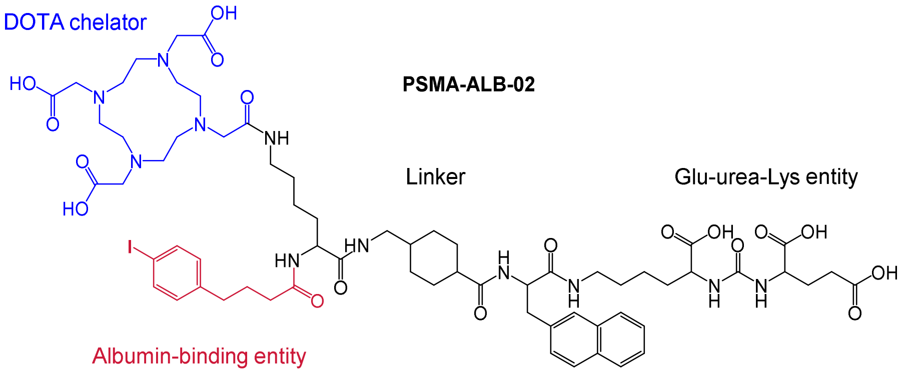

Figure S12. Chemical structure of PSMA-ALB-02 consisting of a DOTA-chelator (blue), an albumin-binding entity (red) and a glutamate-urea-lysine (Glu-urea-Lys)-based PSMA-binding entity.

A representative HPLC chromatogram of the ^{44}Sc-PSMA-ALB-01 is shown in Figure S13.

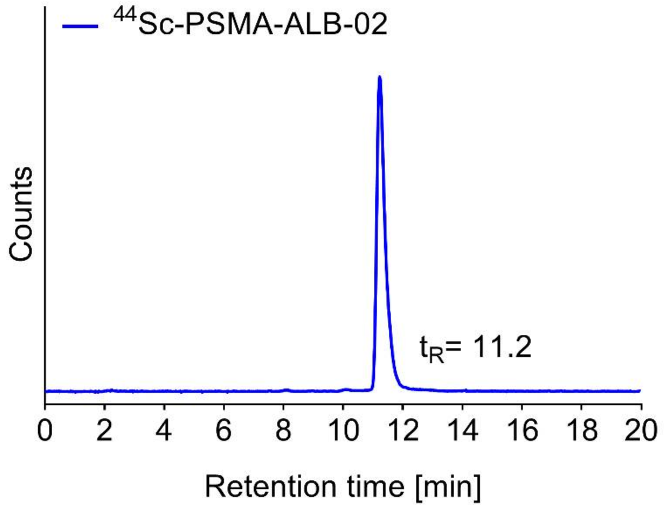

Figure S13. Representative chromatogram of ^{44}Sc-PSMA-ALB-02, demonstrating the product peak and its retention time in minutes (t_R = 11.2 min).

6.2 Biodistribution Studies in Tumor-Bearing Mice

^{44}Sc-PSMA-ALB-02 (5 MBq, 1 nmol, 100-200 μL) was injected in the tail vein of tumor-bearing mice. Mice were sacrificed at 1 h and 4 h post injection (p.i.) of ^{44}Sc-PSMA-ALB-02 (n = 3). Selected tissues and organs were collected, weighed and measured using a γ-counter (Perkin Elmer, Wallac Wizard 1480). The results were decay-corrected and listed as a percentage of the injected activity per gram of tissue mass (% IA/g). In line with the concept of the albumin-binding properties with PSMA-ALB-02, the initial blood activity level after injection of ^{44}Sc-PSMA-ALB-02 was, at 19 % IA/g at 1 h p.i., relatively high but decreased to 7 % IA/g at 4 h p.i.. Fast accumulation of activity was observed in the PSMA-positive PC-3 PIP tumor (47 % IA/g; 1 h p.i.), which increased further over time and

almost doubled (87 % IA/g; 4 h p.i.) over the following hours of distribution (Figure 9). The uptake in PSMA-negative PC-3 flu tumors was even low at both time points of investigation (6-7 % IA/g). The same held true for other non-targeted organs and tissues, including the liver and muscles. The uptake of ^{44}Sc-PSMA-ALB-02 in the kidneys was high (77 ± 10 % IA/g) at 1 h p.i. and slowly cleared over the first few hours after injection (58 ± 6 % IA/g; 4 h p.i) (Figure S14).

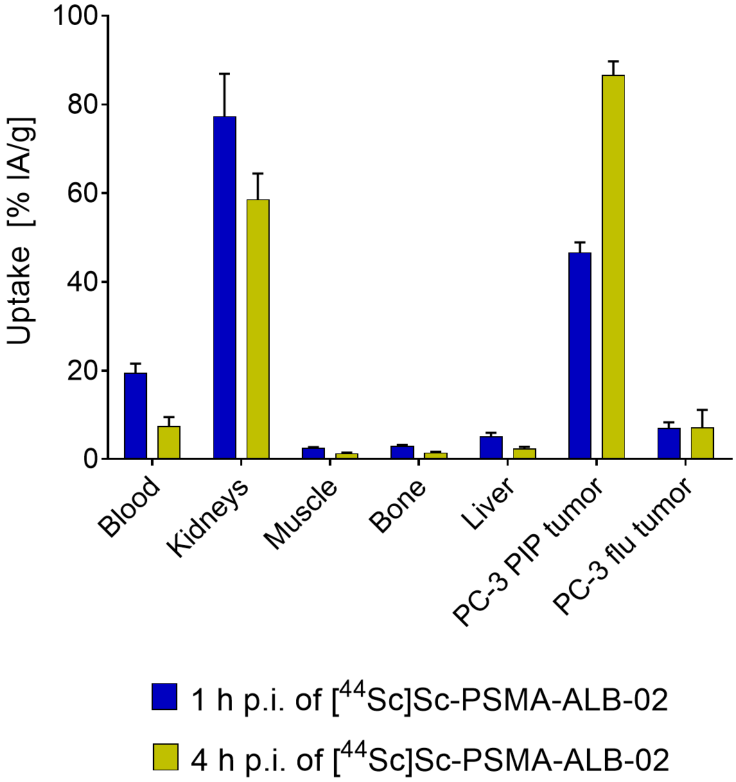

Figure S14. Biodistribution data obtained 1 h and 4 h after injection of [^{44}Sc]Sc-PSMA-ALB-02 in PC-3 PIP/flu tumor-bearing nude mice. Values are indicated as average ± SD (n = 3).

7. **References**

1. Morales-Flórenz V., Santos A., Romero-Hermida, I., Esquivias L. Hydration and carbonation reac-tions of calcium oxide by weathering: kinetics and changes in the nanostructure, Chem. Eng. J. 2015, 265, 194-200.
2. T. S. Carzaniga, Study of Scandium Radio-isotope Production for Theranostics with medical Cyclo-trons, PhD Thesis, University of Bern, 2019.
3. Sinha M. K., Pramanik S., Kumari A., Sahu S. K. et al. Recovery of value added products of Sm and Co from waste SmCo magnet by hydrometallugical route, Sep. Purif. Technol. 2017, 179, 1-12.
4. Pourmand A., Daupphas N. Distribution coefficients of 60 elements on TODGA resin: Application to Ca, Lu, Hf, U and Th isotope geochemistry, Talanta 2010, 81, 741-753.

© 2020 by the authors. Submitted for possible open access publication under the terms and conditions of the Creative Commons Attribution (CC BY) license (http://creativecommons.org/licenses/by/4.0/).
